# The quantum theory of time: a calculus for q-numbers

**DOI:** 10.1098/rspa.2021.0970

**Published:** 2022-07

**Authors:** Samuel Kuypers

**Affiliations:** The Clarendon Laboratory, University of Oxford, Oxford OX1 3PU, UK

**Keywords:** quantum theory, Page–Wootters, time, Heisenberg picture, q-number calculus

## Abstract

In quantum theory, physical systems are usually assumed to evolve relative to a c-number time. This c-number time is unphysical and has turned out to be unnecessary for explaining dynamics: in the timeless approach to quantum theory developed by Page & Wootters 1983 *Phys. Rev. D* **27**, 2885. (doi:10.1103/PhysRevD.27.2885), subsystems of a stationary universe can instead evolve relative to a ‘clock', which is a quantum system with a q-number time observable. Page & Wootters formulated their construction in the Schrödinger picture, which left open the possibility that the c-number time still plays an explanatory role in the Heisenberg picture. I formulate their construction in the Heisenberg picture and demonstrate how to eliminate c-number time from that picture, too. When the Page–Wootters construction is formulated in the Heisenberg picture, the descriptors of physical systems are functions of the clock's q-number time, and derivatives with respect to this q-number time can be defined in terms of the clock's algebra of observables, which results in a calculus for q-numbers.

## 1. Introduction

It is a principle of quantum theory that all descriptors^1^ of physical systems are q-numbers^2^. For example, the momentum and position of a particle are canonically conjugate q-numbers; the descriptors of a qubit adhere to the Pauli algebra, and the descriptors of fermions are Grassmann operators. Due to its significance, I shall call this the *fundamental principle of quantum theory*.

Because of this fundamental principle, time has a peculiar status within quantum theory: time is a c-number, and so it cannot be the descriptor of a physical system. This c-number time is, instead, an unphysical backdrop against which quantum systems can change. In fact, c-number time is all but identical to Newton's absolute time [1], which

*of itself, and from its own nature flows equably without regard to anything external, and by another name is called duration: relative, apparent, and common time, is some sensible and external (whether accurate or unequable) measure of duration by the means of motion…*

In other words, c-number time ‘flows' independently of the behaviour of quantum systems, and it should not be confused with clock time—or what Newton called a *measure of duration by the means of motion*.

To make clear the distinction between clock time and c-number time, consider a hypothetical situation in which all physical systems in the universe suddenly ‘freeze' relative to c-number time.^3^ Clocks are physical systems and would be stationary during this ‘freeze', which makes the ‘freeze' and its duration completely undetectable to any observer in the universe. C-number time must therefore be just as unmeasurable as the duration of the ‘freeze'.

C-number time is unphysical because it *‘*flows without regard to anything external'. Yet, clock time is physical: it is a descriptor of a clock, which is a physical system. Clock time is also measurable: it can be straightforwardly measured, by the usual methods, on the clock.

In classical physics, it is therefore possible to make time manifestly physical by replacing Newton's absolute time with clock time. For example, Barbour [3] has shown that a universe consisting solely of classical systems that interact with one another through Newtonian gravity can give rise to clocks and that the dynamical laws of such a universe can be formulated entirely in terms of clock time.

Similarly, Page & Wootters [4] demonstrated how quantum theory's c-number time can be replaced by clock time. They did this by proposing a quantum model of a completely stationary universe. This universe is stationary because its state-vector |Ψ*_S_*〉 is an eigenstate of the universe's Hamiltonian so that no measurable quantity of this model universe will depend on c-number time. Hence, it is as if the c-number time is not there at all. Within this stationary universe, systems can instead change relative to one another. In particular, they can change relative to a quantum clock, which has an associated q-number known as the *time observable* that completely replaces the role of c-number time. So, the Page–Wootters construction resolves the conflict between c-number time and the fundamental principle of quantum theory—at least in the Schrödinger picture.

However, it is not clear that the Page–Wootters construction eliminates the need for c-number time from the Heisenberg picture because, although the Heisenberg and Schrödinger pictures make identical predictions, they are conceptually different formulations of quantum theory. One such difference is that in the Heisenberg picture, the time-varying entities are the system's q-number descriptors, which affect each other only via local interactions, whereas the Schrödinger-picture state vector is non-local [5]. And even when a system's Schrödinger-picture state vector is time-invariant, that same system can have Heisenberg-picture descriptors that nonetheless depend on c-number time (see §3). If c-number time plays an explanatory role in the Heisenberg-picture Page–Wootters construction, the conflict with the fundamental principle of quantum theory would not have been resolved after all, at least not in that picture.

Suppose that the Page–Wootters construction cannot be adequately formulated in the Heisenberg picture—e.g. perhaps the Heisenberg picture cannot validly describe the entire universe. In that case, the Schrödinger picture would be the more fundamental description of quantum theory. This would be problematic since there are good reasons to regard the Heisenberg picture as not less but *more* fundamental than the Schrödinger picture. For instance, unlike the Schrödinger picture, the Heisenberg picture is a local description of quantum systems [5,6].^4^ If the Heisenberg picture is the more fundamental description of quantum theory, it should admit a formulation of the Page–Wootters construction that is independent of c-number time.

In this paper, I demonstrate that the Page–Wootters construction eliminates the need for c-number time in both the Schrödinger and Heisenberg pictures. When the Page–Wootters construction is formulated in the Heisenberg picture, the descriptors of physical systems turn out to be functions of the clock's q-number time observable. Moreover, one can define derivatives with respect to q-number time in terms of the clock's algebra of observables so that it is possible to refer solely to q-number time in formulating equations of motion. The theory of q-number functions and their derivatives is what I will call the *q-number calculus*.^5^

## 2. The Page–Wootters construction in the Schrödinger picture

In the Page–Wootters construction, the universe is modelled as a non-relativistic system that evolves according to the Schrödinger equation. To make c-number time irrelevant, Page & Wootters assumed that the universe is stationary. In the Schrödinger picture, this means the universal state-vector |Ψ*_S_*〉 is an eigenstate of the universe's Hamiltonian $H_{S}$,

2.1 $${\hat{H}}_{S}|\Psi_{S}\rangle =0.$$

Here and throughout, I use the subscript *S* to indicate that a q-number or a vector is part of the Schrödinger picture. Due to (2.1), it now appears as if the Page–Wootters universe is completely timeless, but subsystems of this universe can nonetheless evolve relative to one another.^6^ In particular, they can evolve relative to a clock.

In the Page–Wootters construction, a clock C is a quantum system with the constitutive property that it possesses a pair of descriptors ${\hat{h}}_{S}$ and ${\hat{t}}_{S}$ that are canonically conjugate

2.2 $$[{\hat{t}}_{S},{\hat{h}}_{S}]=i\hat{1}.$$

ℏ has been set equal to 1 throughout this paper. The q-numbers ${\hat{h}}_{S}$ and ${\hat{t}}_{S}$ live on a Hilbert space ***H****_C_*, and $\hat{1}$ denotes the unit observable on that space. ${\hat{t}}_{S}$ is the clock's *time observable*, so named because the eigenstates of ${\hat{t}}_{S}$ represent different times—i.e. the eigenstate |t⟩ of ${\hat{t}}_{S}$ with eigenvalue *t* has the physical meaning ‘it is clock time *t*’.

The q-number ${\hat{h}}_{S}$ generates translations of ${\hat{t}}_{S}$: by using the algebraic relation (2.2), one can readily verify that an eigenstate |*t*〉 of ${\hat{t}}_{S}$ is shifted by a c-number factor *λ* when it is multiplied by the translation operator $e^{-i{\hat{h}}_{S}\lambda }$, i.e.

2.3 $$e^{-i{\hat{h}}_{S}\lambda }|t\rangle =|t+\lambda \rangle ,$$

and if *λ* is taken to be infinitesimally small, it follows from (2.3) that

2.4 $${\hat{h}}_{S}|t\rangle =i\frac{\text{d}}{\text{d}t}|t\rangle .$$

Certain properties of the clock are invariant for all its states. One such property, the constitutive algebra (2.2), we have already seen. A second invariant property is the spectrum of eigenvalues of ${\hat{t}}_{S}$, denoted $\text{Sp}({\hat{t}}_{S})$. The spectrum $\text{Sp}({\hat{t}}_{S})$ is equal to R because (2.3) implies that if some real number *t* is an eigenvalue of ${\hat{t}}_{S}$, then *t* + *λ* must also be an eigenvalue of ${\hat{t}}_{S}$, for any λ∈ R. In a similar vein, the spectrum of ${\hat{h}}_{S}$ can be shown to be equal to R as well.

C functions as an ideal clock if the ‘rest of the universe' evolves according to the Schrödinger equation relative to C, where the ‘rest', denoted ℜ, constitutes the set of quantum systems in the universe that are not the clock. As will be shown below, the state of ℜ evolves according to the Schrödinger equation relative to clock time if C and ℜ are dynamically isolated from one another. Hence, the Hamiltonian of the universe, denoted ${\hat{H}}_{S}$, should contain no interaction terms, so that

2.5 $${\hat{H}}_{S}={\hat{H}}_{S}+{\hat{h}}_{S}.$$

Here, the Hamiltonian of the clock is ${\hat{h}}_{S}$, and ${\hat{H}}_{S}$ is the Hamiltonian of ℜ.

One obtains the state of ℜ relative to the clock being in the state ‘it is clock time *t*’ by taking the partial inner-product of the universal state-vector |Ψ*_S_*〉 and the eigenstate |t⟩ of ${\hat{t}}_{S}$,

2.6 $$|\psi (t)\rangle \overset{\mathrm{def}}{=}\langle t\text{|}\Psi_{S}\rangle \rangle .$$

The 〉〉 show, where necessary, that |Ψ*_S_*〉 resides on a larger Hilbert space than |*t*〉. By using (2.6), (2.5), (2.4) and (2.1), one can now deduce that |*ψ*(*t*)〉 obeys the Schrödinger equation [10],

$$i\frac{\text{d}}{\text{d}t}|\psi (t)\rangle =i\frac{\text{d}}{\text{d}t}\langle t\text{|}\Psi_{S}\rangle \rangle =-\langle t|{\hat{h}}_{S}|\Psi_{S}\rangle \rangle =\langle t|{\hat{H}}_{S}|\Psi_{S}\rangle \rangle ={\hat{H}}_{S}|\psi (t)\rangle .$$

Thus, the eigenvalues of ${\hat{t}}_{S}$ correspond to the time-parameter of the ‘rest' of the universe, so one can formulate dynamics in terms of clock time, despite the universe being stationary with respect to c-number time.

If a moment such as |*ψ*(*t*)〉|*t*〉 is likened to a snapshot, the universal state-vector |Ψ*_S_*〉 is like a photo album containing the totality of such snapshots. To put this less metaphorically and more mathematically, the universal state-vector is the integral of the relative states over all possible clock times

$$|\Psi_{S}\rangle =\int_{-\infty }^{\infty }|\psi (t)\rangle |t\rangle \text{d}t.$$

This way of formulating the Page–Wootters construction shows its deep connection to Everett's relative-state formalism [11]: the state |*ψ*(*t*)〉|*t*〉 is a relative state, representing the state of the universe |Ψ*_S_*〉 relative to the clock being in the state |*t*〉. Due to the role that Everett's relative-state formalism plays in the Page–Wootters construction, different times are equivalent to different Everett universes [12].^7^

Page & Wootters' achievement is that they constructed a quantum theory of clocks and that in their universe, all change must be change relative to such clocks. In this way, the unphysical c-number time is made completely unnecessary for explaining change and dynamics, which solves the conflict between c-number time and the fundamental principle of quantum theory.

## 3. Q-number time in the Heisenberg picture

Moving between the Heisenberg and Schrödinger pictures is typically a straightforward procedure that consists of applying unitaries to a system's descriptors and state vector. However, this procedure is not invariably straightforward: because of conceptual difficulties, Everett's relative-state formalism has only recently been translated to the Heisenberg picture [13]. And since Everett's relative-state formalism is essential to formulating the Page–Wootters construction, similar issues also appear in translating that construction to the Heisenberg picture.

One such issue is that the Heisenberg descriptors can still depend on c-number time even if the Schrödinger-picture state vector is time-invariant. Consider, for instance, a qubit Q in the Heisenberg picture. Following [5], the state of the qubit at c-number time *λ* is represented by a fixed Heisenberg state and a triple of q-number descriptors,

$$\hat{q}(\lambda )=({\hat{q}}_{x}(\lambda ),\;{\hat{q}}_{y}(\lambda ),\;{\hat{q}}_{z}(\lambda )),$$

that satisfy the Pauli algebra at all times *λ*

$$\begin{array}{l} {\hat{q}}_{i}(\lambda ){\hat{q}}_{j}(\lambda )=\delta_{ij}\hat{1}+i\epsilon_{ij}^{\;\;k}{\hat{q}}_{k}(\lambda )\\ \;{\left({\hat{q}}_{i}(\lambda )\right)}^{†}={\hat{q}}_{i}(\lambda ) \end{array}\}\;(i,j,k\in \{x,y,z\}).$$

Here, † denotes Hermitian conjugation. Throughout this paper, I shall use Einstein's summation convention by summing over indices that appear twice in a product, so in the expression above, *k* is summed over all its possible values {*x*, *y*, *z*}. If Q is an isolated system, its descriptors $\hat{q}(\lambda )$ evolve unitarily and satisfy the Heisenberg equation of motion

3.1 $$\begin{array}{r} \frac{d\hat{q}(\lambda )}{d\lambda }=i[\hat{H},\hat{q}(\lambda )], \end{array}$$

where $\hat{H}$ is the qubit's Hamiltonian.

In the Schrödinger-picture Page–Wootters construction, *λ* is irrelevant to the system's description because the universal state-vector is invariant with respect to *λ*. Yet, in the Heisenberg picture, even when the expectation values of the system do not depend on *λ*, the descriptors $\hat{q}(\lambda )$ will in general depend on c-number time. For instance, the equation of motion (3.1) will be formulated in terms of *λ*, and the descriptors will be functions of *λ*, regardless of their expectation values. Thus, c-number time apparently plays a more fundamental role in the Heisenberg picture than it does in the Schrödinger picture.

But it need not play any role. The Heisenberg equation of motion of the Page–Wootters universe can be formulated entirely in terms of q-number time. And because such a formulation is possible, c-number time can be all but expunged from the Heisenberg picture. To formulate an equation of motion in terms of the q-number time $\hat{t}$, I shall first need to describe, in the Heisenberg picture, what a function of $\hat{t}$ is and how to define derivatives of such functions. Functions of $\hat{t}$ can be defined in terms of the set of *possible states* that a clock can be in.

### (a) Possible states

Physical systems are defined by a set of possible states—‘possible' in the counterfactual sense that these are the states that the system *could be in*, regardless of the dynamics of the system. For example, consider a qubit whose state is represented by a Heisenberg state |Ψ⟩ and a triple of time-invariant descriptors $\hat{q}$ that satisfy the Pauli algebra. For fixed |Ψ⟩, this qubit's set of possible states can be defined as follows: $P\overset{\mathrm{def}}{=}\{U^{†}\hat{q}U:U^{†}U=UU^{†}=\hat{1}\}$, where the unitary *U* is a function solely of $\hat{q}$.

Physical systems also have a *history*, which is a one-parameter family of states uniquely specified by the system's equations of motion and initial conditions. For a fixed Heisenberg state, the qubit's history H is a set of triples $\hat{q}(\lambda )\in P$ that depend on a c-number time *λ* and adhere to the qubit's equation of motion. The parameterized $\hat{q}(\lambda )$ must also satisfy an initial condition so that the qubit's history H is a parameterized curve on P.

There is a key difference between the time-dependent $\hat{q}(\lambda )$ in H and the more fundamental descriptors $\hat{q}$ in P: the triple $\hat{q}$ is part of the counterfactual set of states that the system *could be in*, independently of which states the system *will actually be in* according to its dynamical laws and initial conditions. Hence, the triple$\;\hat{q}$ and Heisenberg state |Ψ〉 represent a more fundamental, timeless notion of state. It is this timeless notion of state that I will refer to throughout the following sections unless stated otherwise.

### (b) The possible states of the clock

In the Heisenberg picture, a possible state of the clock C is fully specified by a Heisenberg state |Ψ〉 and two Hermitian descriptors $\hat{t}$ and $\hat{h}$ that are canonically conjugate, as in (2.2). These q-numbers live on a Hilbert space ***H****_C_*, and just like in the Schrödinger picture, the spectrum of eigenvalues of $\hat{t}$ is $\text{Sp}(\hat{t})=R$. The individual eigenvalues of $\hat{t}$ can be analysed through a projection operator ${\hat{\Pi }}_{t}(\hat{t})$, which has the following property

3.2 $$\begin{array}{r} \hat{t}{\hat{\Pi }}_{t}(\hat{t})=t{\hat{\Pi }}_{t}(\hat{t})\;(\text{for all }t\in \text{Sp}(\hat{t})). \end{array}$$

These projectors sum to unity, making the set $\left\{{\hat{\Pi }}_{t}(\hat{t}):t\in \text{Sp}(\hat{t})\right\}$ a projection-valued measure, in terms of which the q-number $\hat{t}$ has a spectral decomposition, namely

$$\hat{t}=\int_{-\infty }^{\infty }t{\hat{\Pi }}_{t}(\hat{t})\text{d}t.$$

$\hat{t}$ and $\hat{h}$ and all other q-number descriptors of the clock have expectation values that are determined by the Heisenberg state |Ψ〉.^8^ The expectation value of an arbitrary descriptor $\hat{A}$ shall be denoted as $\left\langle \hat{A}\rangle \overset{\mathrm{def}}{=}\langle \Psi |\hat{A}|\Psi \right\rangle$. Furthermore, when this descriptor has the property that $\langle {\hat{A}}^{2}\rangle ={\langle \hat{A}\rangle }^{2}$, the Heisenberg state |Ψ〉 is an eigenstate of $\hat{A}$ with eigenvalue $\left\langle \hat{A}\right\rangle$, and $\hat{A}$ is then said to be *sharp* with that eigenvalue.

A state of the clock for which $\hat{t}$ is sharp with the value *t* has the unambiguous physical meaning ‘it is clock time *t*’, and the clock is then said to be in a *clock state* since it is a state of the clock that represents a definite time*.* When $\hat{t}$ is non-sharp, the clock is in a superposition of clock states; in which case, there is no fact about which time the clock represents. Yet, even when $\hat{t}$ is non-sharp, there is a fact about the time that the clock represents in a *relative state*. That is to say that when $\hat{t}$ is non-sharp, one can consider other systems in the Page–Wootters universe relative to the clock being in the state ‘it is clock time *t*’ by projecting the Heisenberg state onto a *relative Heisenberg state* |Ψ*_t_*〉, which is defined as follows:

$$|\Psi_{t}\rangle \overset{\mathrm{def}}{=}\frac{{\hat{\Pi }}_{t}(\hat{t})|\Psi \rangle }{\sqrt{\left\langle {\hat{\Pi }}_{t}(\hat{t})\right\rangle }},$$

where the factor $\sqrt{\left\langle {\hat{\Pi }}_{t}(\hat{t})\right\rangle }$ in the denominator is assumed to be non-zero and ensures that the relative Heisenberg state is normalized [13]. The relative Heisenberg state |Ψ*_t_*〉 is an eigenstate of $\hat{t}$ with eigenvalue *t*, so with respect to |Ψ*_t_*〉, the clock will read ‘it is clock time *t*’. Thus, even if $\hat{t}$ is non-sharp and there is no fact about what time the clock represents with respect to the absolute state of the system, the clock can represent a definite time within a relative state.

### (c) Q-number functions

Because of the fundamental principle of quantum theory, c-number time should play no explanatory role in quantum theory. Therefore, a system's equations of motion should be formulated in terms of a q-number time $\hat{t}$, and a solution to such an equation of motion must be a function of $\hat{t}$. In this section, I define what a function of $\hat{t}$ is.

The q-number $\hat{t}$ has neither a largest nor a smallest eigenvalue on the full Hilbert space ***H****_C_*: it is unbounded. And its unboundedness results in conceptual difficulties: for instance, because $\hat{t}$ is unbounded, it would be difficult to define absolute convergence of a series in $\hat{t}$. It is convenient to rely instead on the operator $e^{i\omega \hat{t}}$, where *ω* is a real number with units of frequency so that the product $\omega \hat{t}$ is dimensionless. The q-number $e^{i\omega \hat{t}}$ is bounded—its operator norm is unity. So, in this paper, I shall analyse q-number functions of the form

3.3 $$f(\hat{t})\overset{\mathrm{def}}{=}\overset{N}{\underset{n=-N}{\sum }}a_{n}e^{i\omega_{n}\hat{t}}.$$

*N* is an integer so that (3.3) is a finite sum of the bounded q-numbers $\left\{e^{i\omega_{n}\hat{t}}\right\}$.^9^ Functions of the form (3.3) are therefore automatically bounded q-numbers, as well. To guarantee that a function $f(\hat{t})$ is Hermitian, I assume that its coefficients {*a_n_*} are complex numbers with the property that $a_{n}^{†}=a_{-n}$, and that the frequencies {*ω_n_*} are real numbers with the property that *ω*_−_*_n_* = −*ω_n_*, where − *N* ≤ *n* ≤ *N*. By virtue of being Hermitian, $f(\hat{t})$ has a spectrum of eigenvalues, denoted $\text{Sp}(f(\hat{t}))$.

(3.3) does not represent the most general possible function of $\hat{t}$ since (3.3) does not include a convergent series of q-numbers (see for instance [14]). But for the purposes of this paper, sums of the form (3.3) are sufficiently general because such q-number functions can be used to study the evolution of finite systems. As I shall show in §4, the evolution of a qubit can, for example, be described in terms of the functions $\cos(\omega \hat{t})\;\overset{\mathrm{def}}{=}\;\frac{1}{2}(e^{i\omega \hat{t}}+e^{-i\omega \hat{t}})$ and $\sin(\omega \hat{t})\;\overset{\mathrm{def}}{=}\frac{1}{2i}(e^{i\omega \hat{t}}-e^{-i\omega \hat{t}})$.

The descriptors of physical systems appear to be functions of c-number time, yet fundamentally all time-dependent functions should be functions of $\hat{t}$. If that is so, how can one account for the appearance of c-number functions? The solution to this problem is as follows: the spectral decomposition of an arbitrary function $f(\hat{t})$ is

3.4 $$f(\hat{t})=\int_{-\infty }^{\infty }f(t){\hat{\Pi }}_{t}(\hat{t})\text{d}t,$$

where *f*(*t*) is identical to the sum in (3.3) with $\hat{t}$ replaced by *t*. So a measurement of $f(\hat{t})$ will yield a measurement result that is equal to an eigenvalue $f(t)\in \text{Sp}(f(\hat{t}))$. One can therefore account for the appearance of a c-number function *f*(*t*) by referring solely to the q-number function $f(\hat{t})$ and its eigenvalues.

### (d) Q-number derivatives

$f(\hat{t})$ belongs to a possible state of the clock, and since the clock has so far not been assumed to be subject to any dynamical laws, there is no rate of change of $f(\hat{t})$. Yet, an eigenvalue *f*(*t*) of $f(\hat{t})$ does change relative to $t\in \text{Sp}(\hat{t})$, implying that a derivative of *f*(*t*) is physical meaningful—such a derivative represents the instantaneous rate of change of *f*(*t*) with respect to clock time. Because the eigenvalues of $f(\hat{t})$ have well-defined and physically meaningful derivatives, might it be similarly possible to define a derivative of $f(\hat{t})$ with respect to $\hat{t}$, even in the absence of dynamics*?* In a simplified way, this problem may be summarized as follows:

3.5 $$\text{if}\;\lambda \overset{\;}{\to }\hat{t},\;\text{then}\;\frac{d}{d\lambda }\overset{\;}{\to }?$$

Here, *λ* represents c-number time, and the arrow ‘$\overset{\;}{\to }$’ should be understood to mean ‘is replaced by’.^10^

To solve the problem described in (3.5), one can define a derivative of $f(\hat{t})$ with respect to $\hat{t}$ as follows:

3.6 $$\frac{\text{d}f(\hat{t})}{\text{d}\hat{t}}\overset{\mathrm{def}}{=}\overset{\infty }{\underset{-\infty }{\int }}\frac{\text{d }f(t)}{\text{d}t}{\hat{\Pi }}_{t}(\hat{t})dt.$$

Here, the c-number *f*(*t*) is an eigenvalue of $f(\hat{t})$. Since $f(\hat{t})$ is by definition equal to the expansion shown in (3.3), an alternative expression for (3.6) is

3.7 $$\frac{\text{d}f(\hat{t})}{\text{d}\hat{t}}=\overset{N}{\underset{n=-N}{\sum }}ia_{n}\omega_{n}e^{i\omega_{n}\hat{t}}.$$

The expressions (3.7) and (3.6) are equivalent since the eigenvalues of (3.7) are exactly the $\frac{\text{d}f(t)}{\text{d}t}$ that appear in (3.6). Notably, the coefficients *ia_n_ω_n_* in (3.7) satisfy the constraint that ${\left(ia_{n}\omega_{n}\right)}^{†}=ia_{-n}\omega_{-n}$ for all *n*, so the derivative of a Hermitian q-number function is also Hermitian.

It is here that we find a remarkable connection between the algebra of the clock's descriptors and the q-number derivative. For example, the derivative of the q-number $e^{i\omega \hat{t}}$ is

3.8 $$\frac{\text{d}(e^{i\omega \hat{t}})}{\text{d}\hat{t}}=i\omega e^{i\omega \hat{t}},$$

where *ω* is some arbitrary frequency. Because of the commutation relation (2.2), this derivative has an alternative formulation in terms of the following commutator^11^

3.9 $$i[\hat{h},e^{i\omega \hat{t}}]=i\omega e^{i\omega \hat{t}}.$$

Hence, the q-number derivatives of $e^{i\omega \hat{t}}$ can be expressed algebraically in terms of the commutator (3.9). This is a striking and unique feature of the clock and its algebra since if the commutation relation (2.2) were different, the correspondence between the q-number derivative (3.8) and the commutator (3.9) would not hold.

To elaborate on the correspondence between the q-number derivative and the commutation relation (3.9), consider the operator $i[\hat{h},\cdot ]$, where ⋅ denotes the operator's input. This operator is a linear map from q-numbers to q-numbers. The operator is linear in the following sense: for any q-numbers $\hat{A}$ and $\hat{B}$, the result of $i[\hat{h},\cdot ]$ on a linear combination of $\hat{A}$ and $\hat{B}$ is equal to the same linear combination of the operator's result on $\hat{A}$ and $\hat{B}$ separately. That is to say that $i[\hat{h},a\hat{A}+b\hat{B}]=ai[\hat{h},\hat{A}]+bi[\hat{h},\hat{B}],$ where *a* and *b* are real-valued constants. So, under the action of $i[\hat{h},\cdot ]$, the image of an arbitrary q-number function $f(\hat{t})$ is

3.10 $$i[\hat{h},f(\hat{t})]=\overset{N}{\underset{n=-N}{\sum }}ia_{n}\omega_{n}e^{i\omega_{n}\hat{t}}.$$

Manifestly, the right side of (3.10) is equivalent to the right side of (3.7) so that the q-number derivative has a more fundamental algebraic expression, namely

3.11 $$\frac{\text{d}f(\hat{t})}{\text{d}\hat{t}}=i[\hat{h},f(\hat{t})].$$

In fact, it is possible to use the commutator (3.11) as the definition of a derivative with respect to $\hat{t}$ instead of definition (3.6). This has the important advantage that functions that depend on both $\hat{t}$ and $\hat{h}$ will then have well-defined derivatives with respect to the time observable—e.g. the derivative of a function of $\hat{h}$ with respect to $\hat{t}$ would be equal to zero according to (3.11), whereas (3.6) does not specify what the derivative of such a function would be. In what follows, I will therefore use (3.11) as the definition of a derivative with respect to the time observable, from which (3.6) straightforwardly follows as a special case when the derivative is of a function of $\hat{t}$ .

(3.11) is one of the pivotal results of this paper: without referring to the c-number time *λ*, derivatives with respect to $\hat{t}$ can be defined algebraically. This theory of q-number functions and their derivatives is what I will call the *q-number calculus*. Remarkably, the q-number calculus is almost completely concealed in the Schrödinger picture due to that picture's focus on the c-number eigenvalues of descriptors. This obfuscates important questions that are natural to ask in the Heisenberg picture. For instance, one salient question that the current formalism gives rise to is, can the q-number derivative defined in (3.11) be generalized to describe derivatives in a curved space–time? It is not obvious how to formulate that question in the Schrödinger picture.

## 4. Page–Wootters in the Heisenberg picture

In the previous section, I demonstrated that the clock's descriptors can be used to formulate a calculus for q-numbers. But how can the descriptors of other physical systems have equations of motion formulated in terms of $\hat{t}$, which is a descriptor that belongs to the clock? To address this issue, consider a Page–Wootters universe that consists of a clock C and a qubit Q. In order to formulate the qubit's dynamics in terms of q-number time, let the qubit be represented by a triple of descriptors that are functions of $\hat{t}$

4.1 $$\hat{q}(\hat{t})=({\hat{q}}_{x}(\hat{t}),\;{\hat{q}}_{y}(\hat{t}),\;{\hat{q}}_{z}(\hat{t})),$$

which satisfy the Pauli algebra

4.2 $$\begin{array}{l} {\hat{q}}_{i}(\hat{t}){\hat{q}}_{j}(\hat{t})=\delta_{ij}\hat{1}+i\epsilon_{ij}^{\;\;k}{\hat{q}}_{k}(\hat{t})\\ \;{\left({\hat{q}}_{i}(\hat{t})\right)}^{†}={\hat{q}}_{i}(\hat{t}) \end{array}\}\;(i,j,k\in \{x,y,z\}).$$

Here, $\hat{1}$ is the unit observable of the composite system. It is not clear from (4.1) and (4.2) alone that $\hat{q}(\hat{t})$ is a function of $\hat{t}$, or anything of the sort, since all that is currently known about these descriptors is that they obey the Pauli algebra. Thus, let the qubit's descriptors be defined as follows:

4.3 $${\hat{q}}_{j}(\hat{t})\overset{\mathrm{def}}{=}\overset{N}{\underset{n=-N}{\sum }}{\hat{A}}_{j,n}e^{i\omega_{n}\hat{t}}\;(j\in \{x,y,z\}),$$

where the coefficients are bounded q-numbers, and *N* is an integer so that the sum is automatically bounded, too. I require that the coefficients $\left\{{\hat{A}}_{j,n}\right\}$ commute with $\hat{t}$ to ensure that the order of the products in (4.3) is unimportant; I will also assume that those coefficients commute with $\hat{h}$, so derivatives of $\hat{q}(\hat{t})$ with respect to $\hat{t}$ can be defined exactly as in (3.11) and (3.6). Furthermore, the qubit's descriptors are Hermitian due to (4.2), so it must be that ${\hat{A}}_{j,n}^{†}={\hat{A}}_{j,-n}$ and that *ω*_−_*_n_* = −*ω_n_* for − *N* ≤ *n* ≤ *N* and all *j* ∈ {*x*, *y*, *z*}. Notably, it is possible for the coefficients to be equal to zero for certain values of *n* and *j*.

The triple $\hat{q}(\hat{t})$ commutes with $\hat{t}$, and because of this, $\hat{q}(\hat{t})$ admits the following decomposition in terms of the projectors in the set $\left\{{\hat{\Pi }}_{t}(\hat{t}):t\in \text{Sp}(\hat{t})\right\}$

4.4 $$\hat{q}(\hat{t})=\int_{-\infty }^{\infty }\hat{q}(t){\hat{\Pi }}_{t}(\hat{t})\text{d}t,$$

where $\hat{q}(t)$ is equivalent to the expression (4.3), but with $\hat{t}$ replaced by *t*. In decomposition (4.4), $\hat{q}(t){\hat{\Pi }}_{t}(\hat{t})$ is a triple of *relative descriptors* [13], so named because these descriptors represent the state of the qubit relative to the clock being in the state ‘it is clock time *t*’.^12^ The relative descriptors represent a fully fledged qubit because they satisfy an appropriate form of the Pauli algebra: using (4.2) as well as the identities that $\hat{q}(\hat{t}){\hat{\Pi }}_{t}(\hat{t})=\hat{q}(t){\hat{\Pi }}_{t}(\hat{t})$ and ${\hat{\Pi }}_{t}(\hat{t}){\hat{\Pi }}_{t^{\prime }}(\hat{t})={\hat{\Pi }}_{t}(\hat{t})\delta (t-t^{\prime })$, where *δ*(*t − t^′^*) denotes the Dirac delta function, one finds

$$\begin{array}{c} \left({\hat{q}}_{i}(t){\hat{\Pi }}_{t}(\hat{t})\right)\left({\hat{q}}_{j}(t){\hat{\Pi }}_{t}(\hat{t})\right)=\delta (0)\left(\delta_{ij}{\hat{\Pi }}_{t}(\hat{t})+i\epsilon_{ij}^{\;\;k}{\hat{q}}_{k}(t){\hat{\Pi }}_{t}(\hat{t})\right)\\ \;{\left({\hat{q}}_{i}(t){\hat{\Pi }}_{t}(\hat{t})\right)}^{†}={\hat{q}}_{i}(t){\hat{\Pi }}_{t}(\hat{t}) \end{array}\}\;(i,j,k\in \{x,y,z\}).$$

Here, the term *δ(0)* can be set to 1, as my use of the Dirac delta function is only necessary because the idealized clock has a continuous spectrum: using a countable set of projectors, which reflects the physical situation because the time observable of a real clock has a discrete spectrum, would replace the Dirac delta by the Kronecker one. When *δ*(*0*) is set to 1, ${\hat{\Pi }}_{t}(\hat{t})$ can be interpreted as the ‘relative unit observable' relative to the clock being in the state ‘it is time *t*’.^13^

Since the q-number coefficients in (4.3) commute with $\hat{t}$ and $\hat{h}$, a derivative of $\hat{q}(\hat{t})$ with respect to $\hat{t}$ can be defined precisely as in (3.11) and (3.6), namely

4.5 $$\frac{\text{d}\hat{q}(\hat{t})}{\text{d}\hat{t}}\overset{\mathrm{def}}{=}i[\hat{h},\hat{q}(\hat{t})],$$

so that

4.6 $$\frac{\text{d}\hat{q}(\hat{t})}{\text{d}\hat{t}}=\int_{-\infty }^{\infty }\frac{\text{d}\hat{q}(t)}{\text{d}t}{\hat{\Pi }}_{t}(\hat{t})\text{d}t.$$

The right side of (4.5) is generally non-zero, as $\hat{q}(\hat{t})$ does not necessarily commute with $\hat{h}$ due to $\hat{q}(\hat{t})$'s dependence on $\hat{t}$. Incidentally, although $\hat{h}$ still satisfies the algebraic relation (2.2), it cannot represent the Hamiltonian of the clock since the clock and qubit must have mutually commuting descriptors—I will discuss this issue in §4b.

Thus far, I have not made any reference to c-number time nor specified the Hamiltonian of the composite system. The descriptors $\hat{q}(\hat{t})$**,** $\hat{t}$ and $\hat{h}$ merely represent a possible state of the qubit and clock. Despite this, the triple $\hat{q}(\hat{t})$ has a physically meaningful derivative with respect to $\hat{t}$, so although the model is completely timeless (in that it does not refer to c-number time), one can define equations of motion with respect to clock time. Hence, just as one would typically impose that a system satisfies the Heisenberg equation of motion with respect to c-number time, I instead require that the qubit's descriptors satisfy the Heisenberg equation of motion with respect to clock time. This assumption can be formulated algebraically, as follows:

4.7 $$[\hat{h}-\hat{H},\hat{q}(\hat{t})]=0\Leftrightarrow \frac{\text{d}\hat{q}(\hat{t})}{\text{d}\hat{t}}=i[\hat{H},\hat{q}(\hat{t})].$$

Here, $\hat{H}$ is the qubit's Hamiltonian relative to clock time; the system will in general also have a Hamiltonian that generates translations of c-number time, which can differ from $\hat{H}$, and which is yet to be specified. It will be introduced later, in §4a.

Equation (4.7) demonstrates the central result of this paper: in the Heisenberg picture, one can formulate the equations of motion algebraically in terms of q-number time, making c-number time unnecessary for expressing dynamical laws. And to formulate equations of motion in terms of q-number time, we had to fundamentally reinterpret what functions of time, and derivatives with respect to it, are.

In the Page–Wootters construction, the relative descriptors correspond to the observable states of the qubit: it is impossible to observe $\hat{q}(\hat{t})$, as it represents the entire history of the qubit; one can only observe the qubit at a particular clock time, as represented by the relative descriptors. Therefore, to match observation, the construction must be such that the relative descriptors satisfy the Heisenberg equation of motion with respect to clock time. Because of (4.7), the qubit's relative descriptors automatically satisfy the Heisenberg equation of motion with respect to clock time, as required: by using (4.4), (4.6) and (4.7), one finds that

$$\int_{-\infty }^{\infty }\left(\frac{\text{d}\hat{q}(t)}{\text{d}t}-[\hat{H},\hat{q}(t)]\right){\hat{\Pi }}_{t}(\hat{t})\text{d}t=0,$$

from which it follows that

4.8 $$\frac{\text{d}\hat{q}(t)}{\text{d}t}=i[\hat{H},\hat{q}(t)]\;(\text{for all }t\in \text{Sp}(\hat{t})).$$

As an example of a solution to (4.7), consider the case in which the relative descriptors at clock time *t* = 0 are equal to the Pauli matrices, i.e. $\hat{q}(0)=({\hat{\sigma }}_{x},\;{\hat{\sigma }}_{y},\;{\hat{\sigma }}_{z})$, and the qubit's Hamiltonian is $\hat{H}=\frac{\Omega }{2}{\hat{\sigma }}_{x}$, where Ω is a real-valued constant with units of frequency. Then, a solution to (4.7) is that $\hat{q}(\hat{t})=e^{i\hat{H}\hat{t}}\hat{q}(0)e^{-i\hat{H}\hat{t}}$, which has the explicit expression

$$\hat{q}(\hat{t})=({\hat{\sigma }}_{x},\;{\hat{\sigma }}_{y}\cos(\Omega \hat{t})+{\hat{\sigma }}_{z}\sin(\Omega \hat{t}),\;{\hat{\sigma }}_{z}\cos(\Omega \hat{t})-{\hat{\sigma }}_{y}\sin(\Omega \hat{t})).$$

From this equation, one can reconstruct the coefficients $\left\{{\hat{A}}_{j,n}\right\}$ and the frequencies {*ω_n_*} in (4.3); in particular, one can express the {*ω_n_*} in terms of Ω.

I have here treated a Page–Wootters model in which a qubit evolves relative to a clock. The qubit in this construction can be generalized to isolated systems of any finite size. For instance, instead of a single qubit, one could consider an isolated system of *n* interacting qubits. Because this system of interacting qubits is isolated and its Hilbert space is finite, the descriptors of those qubits will be periodic functions. Each descriptor can therefore be expressed as a finite sum of the form (4.3), where the descriptors of different qubits have potentially different sets of frequencies {*ω_n_*}. One would then impose that the qubits obey a Heisenberg equation of motion with respect to clock time, similar to the equation of motion in (4.7).

### (a) Removing the c-number time

In the previous section, I formulated a qubit's equations of motion in terms of q-number time, demonstrating that c-number time is not necessary for formulating dynamics. Yet it remains an axiom of quantum theory that systems obey the Heisenberg equation of motion with respect to c-number time. For instance, the Page–Wootters universe is an isolated quantum system, and therefore an arbitrary descriptor of the model, denoted $\hat{A}(\lambda )$, must satisfy the equation

4.9 $$\frac{\text{d}\hat{A}(\lambda )}{\text{d}\lambda }=i[\hat{H},\hat{A}(\lambda )],$$

where *λ* represents c-number time, and $\hat{H}$ is the Hamiltonian of the universe. $\hat{H}$ is different from $\hat{H}$ in (4.7) in that $\hat{H}$ generates translations of *λ*. The descriptor $\hat{A}(\lambda )$ is a solution to (4.9) if $\hat{A}(\lambda )=e^{i\lambda \hat{H}}\hat{A}(0)e^{-i\lambda \hat{H}}$, where $\hat{A}(0)$ is the descriptor at *λ* = 0.

The triple $\hat{q}(\hat{t})$ represents a possible state of the qubit, and if one assumes that this triple represents the qubit's initial (*λ* = 0) state, then the state of the qubit at time *λ* is represented by $e^{i\hat{H}\lambda }\hat{q}(\hat{t})e^{-i\hat{H}\lambda }$. Although the equation of motion of the triple $e^{i\hat{H}\lambda }\hat{q}(\hat{t})e^{-i\hat{H}\lambda }$ has a formulation in terms of q-number time, those descriptors also depend on the unphysical *λ*, which therefore still plays an explanatory role in the construction.

To obviate this problem, let $\hat{H}=\hat{h}:$ for this choice of $\hat{H}$, the equation of motion (4.9) reduces to the requirement that, for any descriptor $\hat{A}(\lambda )$ of the model^14^

4.10 $$\frac{\text{d}\hat{A}(\lambda )\;}{\text{d}\lambda }=\frac{\text{d}\hat{A}(\lambda )\;}{\text{d}\hat{t}}.$$

In other words, due to (4.10), the derivative with respect to the unphysical c-number time can be reinterpreted as a derivative with respect to the manifestly physical q-number time. To satisfy constraint (4.10), the descriptors of the Page–Wootters universe are as follows: $e^{i\hat{H}\lambda }\hat{t}e^{-i\hat{H}\lambda }=\hat{t}+\lambda \hat{1}$, and $e^{i\hat{H}\lambda }\hat{q}(\hat{t})e^{-i\hat{H}\lambda }=\hat{q}(\hat{t}+\lambda \hat{1})$**,** while $\hat{h}$ is invariant with respect to *λ*. Using these descriptors, one can generate all other descriptors of the system via addition and multiplication and scaling (multiplication by a real number), so the system's evolution relative to *λ* has been fully specified.

Finally, to avoid the descriptors' expectation values from depending on *λ*, let the Heisenberg state, denoted |Ψ〉, be an eigenstate of $\hat{H}$. Due to this assumption, all expectation values are independent of *λ*: if $\hat{A}(\lambda )$ is an arbitrary *λ*-dependent descriptor, then it follows that $\left\langle \Psi |\hat{A}(\lambda )|\Psi \rangle =\langle \Psi |e^{i\lambda \hat{H}}\hat{A}(0)e^{-i\lambda \hat{H}}|\Psi \rangle =\langle \Psi |\hat{A}(0)|\Psi \right\rangle$ for all *λ*. As a result of the constraints described in this section, c-number time does not play an explanatory role in the Heisenberg-picture Page–Wootters construction: no expectation value of the model depends on it; derivatives with respect to *λ* can be substituted for derivatives with respect to $\hat{t}$; and a system's equations of motion can instead be formulated entirely in terms of $\hat{t}$.

### (b) Moving between stationary pictures

With the Heisenberg-picture Page–Wootters construction completed, it seems timely to reflect on the connections between the Schrödinger and Heisenberg picture variants of the construction. I have summarized these connections in the chart below.

**Table:** 

the Schrödinger picture	correspondence	the Heisenberg picture
$|\Psi_{S}\rangle =\int_{-\infty }^{\infty }|\psi (t)\rangle |t\rangle \text{d}t$	$\overset{\text{absolute }\text{state}}{⟷}$	$\hat{q}(\hat{t})=\int_{-\infty }^{\infty }\hat{q}(t){\hat{\Pi }}_{t}(\hat{t})\text{d}t$
|ψ(t)⟩|t⟩	$\overset{\text{relative }\text{state}}{⟷}$	$\hat{q}(t){\hat{\Pi }}_{t}(\hat{t})$
${\hat{H}}_{S}={\hat{H}}_{S}+{\hat{h}}_{S}$	$\overset{\text{Hamiltonian}}{⟷}$	$\hat{H}=\hat{h}$
$i\frac{\text{d}}{\text{d}t}|\psi (t)\rangle ={\hat{H}}_{S}|\psi (t)\rangle$	$\overset{\text{equation }\text{of }\text{motion}}{⟷}$	$\frac{\text{d}\hat{q}(\hat{t})}{\text{d}\hat{t}}=i[\hat{H},\hat{q}(\hat{t})]$

The Schrödinger- and Heisenberg-picture Page–Wootters constructions are also mathematically equivalent: as Rijavec [16] has demonstrated, if one starts with a Schrödinger-picture Page–Wootters model that consists of a qubit and clock, then by using the unitary transformation $V\overset{\mathrm{def}}{=}e^{-i{\hat{H}}_{S}{\hat{t}}_{S}}$, one obtains the Heisenberg-picture construction as follows:

4.11 $$\begin{array}{l} V^{†}{\hat{h}}_{S}V={\hat{h}}_{S}-{\hat{H}}_{S},\;V^{†}{\hat{t}}_{S}V={\hat{t}}_{S},\;V^{†}\hat{q}(0)V=e^{i{\hat{H}}_{S}{\hat{t}}_{S}}\hat{q}(0)e^{-i{\hat{H}}_{S}{\hat{t}}_{S}}\\ \;V^{†}{\hat{H}}_{S}V={\hat{h}}_{S},\;\;\;V^{†}{\hat{H}}_{S}V={\hat{H}}_{S},\;\;V^{†}|\Psi_{S}\rangle =|\psi (0)\rangle |TL\rangle \end{array}\}.$$

Here, $|TL\rangle \;\overset{\mathrm{def}}{=}\;\int_{-\infty }^{\infty }|t\rangle \text{d}t$ is the so-called ‘time line' state [17]. Similarly, the inverse transformation $V^{†}$ maps the Heisenberg-picture construction to the Schrödinger picture.^15^

As is evident from (4.11), the Heisenberg descriptors of the clock and the qubit are a mixture of their Schrödinger-picture counterparts—e.g. the Hamiltonian of the clock in the Heisenberg picture is equal to ${\hat{h}}_{S}-{\hat{H}}_{S}$. Furthermore, the descriptors of the clock and qubit must commute, so it follows that ${\hat{h}}_{S}-{\hat{H}}_{S}$ commutes with $\hat{q}(\hat{t})$, from which one recovers the equation of motion (4.7).

### (c) Missed but remembered

Because the Heisenberg state is an eigenstate of $\hat{h}$, the expectation value of each relative descriptor is non-zero—i.e. $\langle {\hat{\Pi }}_{t}(\hat{t})\rangle >0$ for all $t\in \text{Sp}(\hat{t})$*.* Remarkably, this feature does not seem necessary for the Page–Wootters construction to explain time and dynamics [18]. For instance, no observer could detect that the expectation value $\left\langle {\hat{\Pi }}_{t}(\hat{t})\right\rangle$ is identically zero for some interval of $\hat{t}$'s eigenvalues. That is so because, even if certain clock times are ‘missing’ from the Page–Wootters universe, every system in this universe will nonetheless end up in exactly the state it would have been in if those ‘missing' clock times had been there. This is analogous to a situation in the novel *Permutation City* [19], in which a simulated person, Paul, is being rendered by a computer at half-second intervals and not at intermediate moments:

*Paul counted – and the truth was, he felt no different … And that made sense [because] every half-second, his brain was ending up in exactly the state it would have been in if nothing had been left out.*

Perhaps Paul's predicament is like our own in that, in our universe—without our being aware of it—certain moments are ‘missing', despite us having memories of them.

## 5. Open problems in the Page–Wootters construction

The clock in the Page–Wootters universe is ideally accurate: it has infinitely many distinguishable states (i.e. it has infinitely many eigenstates), and so there is no limit on how accurately it can keep track of time. However, this also implies that one can store an infinite amount of information in the clock [20], despite it being widely believed that only a finite amount of information can be stored in any compact region of space due to Bekenstein's bound [21]. It therefore seems that the universe should contain a multitude of unideal clocks, which must be finite physical systems.^16^

That a menagerie of clocks can give rise to something like a global time at all should be due to those clocks being synchronized [10] . To understand what it means for clocks to be synchronized, consider two quantum clocks $C_{1}$ and $C_{2}$, with respective pairs of canonically conjugate descriptors $\left({\hat{t}}_{1},{\hat{h}}_{1}\right)$, and $\left({\hat{t}}_{2},{\hat{h}}_{2}\right)$, where the descriptors of $C_{1}$ commute with those of $C_{2}$.^17^ The clock $C_{2}$ is perfectly synchronized with $C_{1}$ if the observable ${\hat{t}}_{1}-{\hat{t}}_{2}$ is sharp with value zero because, in that case, if one measures ${\hat{t}}_{1}$ and ${\hat{t}}_{2}$ separately and subtracts the two measurement results, zero offset will be detected between the clocks.

These synchronized clocks bear striking similarities to the atlas of a manifold. An atlas consists of a family of charts, which are functions from the manifold to $R^{n}$. A chart resembles an individual clock in that an ideal clock, like $C_{1}$, provides a function from ${\hat{t}}_{1}$ to the continuum through its spectrum, i.e. $\text{Sp}({\hat{t}}_{1})=R$. Furthermore, the eigenvalues $\text{Sp}({\hat{t}}_{1})$ represent clock time, much like how the chart represents coordinates on the manifold. Secondly, the requirement that ${\hat{t}}_{1}-{\hat{t}}_{2}$ is sharp with value zero allows us to determine how clock time ${\hat{t}}_{1}$ is related to clock time ${\hat{t}}_{2}$. This is similar to how the coordinates of one chart can be related to the coordinates of another via a transition map, which is a homeomorphism between two charts in a region where those charts overlap.

Because of these connections, I conjecture that the properties of a space–time manifold can be formulated in terms of q-numbers. Formulating space–time manifolds in this way *prima facie* requires an extension of the Page–Wootters model—for instance, the model should incorporate space [23]. Furthermore, in both special and general relativity, the structure of space–time imposes severe restrictions on the behaviour of clocks: absolute simultaneity is not permitted by special and general relativity, which implies that two space-like separated clocks cannot be synchronized in all reference frames. Likewise, due to the effects of time dilation, clocks will stop ‘ticking' in lockstep when they start moving relative to one another. In a more general Page–Wootters construction, it seems that clocks such as $C_{1}$ and $C_{2}$ should adhere to such restrictions. I shall leave these questions to be addressed in future research.

## Data Availability

This article has no additional data.

## Footnotes

1 The descriptors of a physical system are mathematical objects—e.g. tensors, vectors, real numbers, integers—that represent the state of the system.

2 Q-numbers are numbers that satisfy a non-commutative algebra; they are conventionally represented by operators or Hermitian matrices on a Hilbert space. C-numbers satisfy a commutative algebra—for example, complex numbers are c-numbers. In quantum theory, a system's state is specified by a set of q-numbers and a state vector. For instance, a qubit has *x*-, *y*- and *z*-observables (which are q-numbers), and the state of the qubit is represented by those q-numbers and their expectation values.

3 This thought experiment is based on Shoemaker's [2] discussion of Newton's absolute time.

4 That is to say that only the Heisenberg picture satisfies Einsteinian locality, which requires that ‘the real factual situation of the system *S*_2_ is independent of what is done with the *S*_1_, which is spatially separated from the former’ ([7], p. 85)*.*

5 Dirac [8] was perhaps the first person to conceive of using the Heisenberg picture to formulate a calculus for q-numbers. I shall expand on Dirac's ideas in later sections by clarifying the notion of a q-number function and its derivatives.

6 The Page–Wootters construction is a block universe theory: it is a theory of a stationary system—a ‘block’—in which the future and the past exist ‘simultaneously’. Perhaps the earliest iteration of the block universe theory was invented over two millennia ago, around 500 BC, by the pre-Socratic Greek philosopher Parmenides. Parmenides expounded that the universe is fundamentally unchanging; that all movement is only apparent; and that through the use of reason one can discover that change is an illusion [9]. Parmenides' block universe is remarkably similar to the Page–Wootters construction, as each describes a stationary universe in which time and dynamics are emergent.

7 For C to be an ideal clock, there can be no interactions between it and the rest of the universe. Hence, if one were to measure C, it would cease to be an ideal clock. So, time can only be measured imperfectly in the Page–Wootters construction. This does not affect other features of the construction—for instance, the universal state vector can nonetheless be decomposed into relative states (relative to clock time) because the clock and the rest of the universe need only be entangled for that.

8 Other observables of the clock are for instance ${\hat{t}}^{2}$ and ${\hat{h}}^{2}$, and their expectation values can in general not be deduced from those of $\hat{t}$ and $\hat{h}$.

9 The frequencies $\left\{\omega_{n}\right\}$ are c-number functions of *n*, and their expressions will in practice be determined by a system's equations of motion, as shall be discussed in §4.

10 This problem is related to the problem of formulating quantum theory without referring to classical concepts, as discussed by Deutsch [15]*.*

11 The identity (3.9) can be derived as follows: because of the commutation relation (2.2), the unitary $e^{i\omega \hat{t}}$ is a translation operator of $\hat{h}$ so that $e^{-i\omega \hat{t}}\hat{h}e^{i\omega \hat{t}}=\hat{h}+\omega \hat{1}$, which can be rearranged to give the desired identity.

12 These are the relative descriptors relative to the clock states ‘it is clock time *t*’ since a projector ${\hat{\Pi }}_{t}(\hat{t})$ projects onto a Heisenberg state for which $\hat{t}$ is sharp with value *t* (see §3*b*).

13 Because both $\hat{q}(t){\hat{\Pi }}_{t}(\hat{t})$ and $\hat{q}(t)$ satisfy the same algebra, the inclusion of the projector is not always relevant. So as an abuse of terminology, I shall refer to both $\hat{q}(t){\hat{\Pi }}_{t}(\hat{t})$ and $\hat{q}(t)$ as the relative descriptors of the qubit, relative to clock time *t*.

14 Equation (4.10) is satisfied by all descriptors of the model because the commutator (3.11) is the definition of a derivative with respect to $\hat{t}$, so (3.11) is well-defined for all descriptors. Notably, (3.6) is a special case of (3.11) when the derivative is of a descriptor that commutes with $\hat{t}$.

15 This transformation also allows us to incorporate time-dependent Hamiltonians into the formalism presented here. For instance, if the Hamiltonian of the qubit contains explicit time-dependence, denoted as $\hat{H}(\hat{t})$, then the transformation that maps the Schrödinger picture to the Heisenberg picture will be a time-ordered exponential of $\hat{H}(\hat{t})$; see for instance [17].

16 An unideal clock must have a time observable with finitely many eigenvalues, so such an observable represents discrete time. In that case, one should be able formulate the equations of motion of the rest of the universe as difference equations, where for comparatively large systems, these difference equations will be approximately identical to the Heisenberg equation (4.7). I shall leave the explicit formulation of a Heisenberg Page–Wootters construction with unideal clocks for future research. For more on how to use finite physical systems as clocks, see the work of Smith & Ahmadi [22] and Rijavec [16].

17 Notably, $C_{1}$ and $C_{2}$ are ideal clocks because they have canonical conjugate observables that necessarily live on an infinite dimensional Hilbert space. Although such ideal clocks cannot be physical, studying them will nonetheless be informative since it will tell us how well two physical clocks can be synchronized in the absence of other limitations.
